# Optimizing of the extraction conditions for anthocyanin’s from purple corn flour (*Zea mays* L): Evidences on selected properties of optimized extract

**DOI:** 10.1016/j.fochx.2022.100521

**Published:** 2022-11-23

**Authors:** Mioara Gabriela Slavu Ursu, Ștefania Adelina Milea, Bogdan Păcularu-Burada, Loredana Dumitrașcu, Gabriela Râpeanu, Silvius Stanciu, Nicoleta Stănciuc

**Affiliations:** Faculty of Food Science and Engineering, Dunarea de Jos University of Galati, 111 Domnească Street, 800201 Galați, Romania

**Keywords:** Purple corn flour, Anthocyanin’s extraction, Response surface methodology, Yeast, Inhibitory activities for tyrosinase, α-Amylase and α-glucosidase inhibitory activity

## Abstract

In this study, the Response Surface Methodology was used to optimize the extraction conditions for anthocyanins from purple corn flour (PCF), whereas the optimized extract was characterized in terms of phytochemicals content, inhibitory activity against metabolic associated enzymes and the potential to stimulate the metabolic activity of yeasts. The optimized conditions using a Box-Behnken design (BBD) of response surface methodology (RSM) were as follow: temperature (39 °C), extraction time (5 h), liquid/solid ratio (30 mL/g) and ethanol concentration (73 %), yielding a maximum response predicted and experimental values of 13.77 and 14.04 ± 0.02 mg cyanidin 3-*O*-glucoside equivalents (C3G)/g dry weight (DW). The extract showed a high content in myricetin, quercetin 3-β-d-glucoside, kaempferol, whereas the main anthocyanin was C3G. The extract was test the effect on metabolic activity of *Saccharomyces cerevisiae*, with the highest multiplication rate obtained at 1.2 mg/100 mL. The extract showed potential antidiabetic, hypocholesterolemic and preventive effects against Parkinson’s disease and melanoma. The findings provide the optimum conditions to obtain an anthocyanin enriched extract, for multiple applications.

## Highlights

• The anthocyanins extraction conditions were optimized using Response Surface Methodology.
• The extract was rich in myricetin, quercetin 3-β-d-glucoside, kaempferol and cyanidin-3-*O*-glucoside.
• The extract had a significant effect on metabolic activity of *Saccharomyces cerevisiae*.
• The extract showed inhibitory activity against tyrosinase, α-amylase, α-glucosidase and lipase.

## Introduction

More attention has been given recently by food producers in elaboration foods with unique sensorial and nutritional characteristics, whereas rich and/or enriched in bioactive compounds content. This trend is determined by the relevance of bioactive compounds, especially polyphenols, in promoting health related benefits. For example, an increasing number of studies have demonstrated various biological activities of anthocyanins such as anticancer, antioxidant, anti-inflammatory, antimutagenic, etc. (Lao et al., 2017). Therefore, significant efforts are made to develop technological alternatives for value-added products, by supplementing with plant-based materials rich in bioactive compounds, whereas making them more appealing to health-conscious population (Betoret & Rosell, 2019).

Purple corn (*Zea mays* L) has been recently named as “superfood”, due to its remarkable content in polyphenols, with potential health benefits (Lee et al., 2021). Purple corn was originally cultivated in Andean region of Peru, however, nowadays is well-known worldwide, especially in Asia, Europe, and the United States (Zhao et al., 2008). The main, individual characteristic of purple corn is the high content of polyphenolic compounds, especially anthocyanins, which is why purple corn culture is agricultural material in the food and pharmaceutical industries (Lao, Sigurdson & Giusti, 2017). Therefore, anthocyanins are the most critical phytochemicals in the purple corn, including cyanidin-3-*O*-dimalonylglucoside, cyanidin-3-*O*-glucoside, pelargonidin-3-*O*-glucoside, peonidin-3-*O*-glucoside, distributed in leaves, cobs, and seeds (Cuevas Montilla et al., 2011). Besides anthocyanins, the purple corn is a rich source of phenolic acids, carotenoids, flavonoids, resistant starch, dietary fiber, minerals, such as phosphorus, potassium and magnesium), vitamins (A, B, E, and K), phytosterols (Siyuan et al., 2018). For example, some health-related relevant compounds were identified by Lee et al. (2021), such as two morphinan alkaloids (sinomenine and codeine), known for their anti-inflammatory and antispasmodic properties (Chu et al., 2018, Shukla et al., 2019). Additionally, Lee et al. (2021) reported the presence of cyclandelate, linoleic and docosahexaenoic acids, tiotropium, bisoprolol, glycocholic acid, 6-gingerol, 4-methylumbelliferone, associated with neuroprotective, anti-inflammatory, anti-nausea, antioxidant, antidiabetic, anticancerigenic activities, while promoting the blood circulation, regulating the blood pressure, controlling cholesterol level, etc. Therefore, due to the abundance of bioactive, the purple corn may be used in different food, pharmaceuticals, nutraceuticals and biotechnology applications, which can significantly improve several lifestyles associated diseases, including obesity, diabetes, hyperglycemia, hypertension and cardiovascular disease. Additionally, anthocyanins are valuable dyes and pigments for other foods, due to their intense variety of blue, purple and red colors, coupled to their lack of toxicity. Currently, the main sources for anthocyanins are given by intense colored fruits and vegetables, such as blueberries, blackthorn, red grapes, black carrots, sweet potatoes etc. and related processing by-products. However, when considering the anthocyanins valorizations, the high costs associated with the extraction have largely limited their use in the food industry and there is a significant interest in exploring the most economical ways of their recovery (Somavat et al., 2016). Therefore, during extraction it is crucial to optimize the ratio of solid–liquid, extraction time and temperature, in order to maximize the extraction capacity, whereas minimizing the extraction time. Secondly, prolonged extraction time (Zhang et al. 2014) or high temperatures (Piyapanrungrueang et al. 2016) may increase the efficiency of anthocyanin extraction, but these conditions also reduce anthocyanin stability. Additionally, some other factors may affect the efficiency of polyphenols extractions, such as the type of solvent, particle size of raw material, liquid to material ratio, solvent concentration, stirring rate, extraction time, extraction temperature, etc.

One of the most used strategies used to maximize the yield of bioactive compound extractions from plant material is Response surface methodology (RSM) (Kamiloglu et al., 2017). The Box-Behnken design (BBD) is a multivariable optimization technique relying on three-level incomplete factorial designs, used for a second-order response surface model (Kaur et al., 2016).

Therefore, the aim of this study was to optimize the extraction of anthocyanins from purple corn flour (PCF) and to test the efficiency of validated extract on the metabolic activity of selected yeast, from the perspectives of using PCF as raw material for bioethanol production and/or adjuvants in food applications. Therefore, considering that ethanol is a cheap and safety solvent, in our study the type of solvent was not investigated. In the first step, the experiments involved testing of four factors, such as time, temperature, liquid/solid ratio and ethanol concentration with the aim of balancing the temperature, the composition of the extraction solution in order to efficiently extract anthocyanins from PCF. Therefore, a four-factor, three-level Box-Behnken design (BBD) with three replicates in the central points was employed, whereas the experimental data were fitted to the quadratic polynomial. Consecutively, the validated extraction model was analyzed, and could provide reference for the large-scale application of polyphenols extraction from PCF. Different analytical procedures were applied to test some selected proprieties of the optimized extracts, such as total polyphenolic content, antioxidant activity and tyrosinase inhibitory activity. The chromatographic analysis was used to identify and quantify the main polyphenolic compounds in the extract. Further, the impact of extract added to yeast culture medium on specific metabolic activity was assessed. Therefore, different concentration of PCF extract was added to bakery yeast culture medium and specific parameters were tested, such as the multiplication, viability of yeast cells and dynamics of alcoholic fermentation.

## Materials and methods

### Plant material

PCF (*Zea mays* L.) was purchased from local producer (Brăila County, Romania) in October 2021. The samples were packed in polyethylene bags and kept at 4 °C until analysis. Moisture of PCF was determined with an air-oven at 135 °C for 2 h (Approved Method 44–19, AACC International, 2010). The purple corn flour used for phytochemicals extraction with a moisture content of <12 % was preliminary sifted through a 100-mesh sieve. The extraction experiments were performed within a maximum storage period of PCF of 30 days.

### Chemicals

Acetonitrile, formic acid, gallic acid, caffeic acid, chlorogenic acid, vanillic acid, *p*-coumaric acid, (-) – epicatechin, myricetin, isorhamnetin, (+) - catechin, cyanidin 3-*O*-glucoside, cyanidin 3-*O*-rutinoside, delphinidin 3-*O*-β-d-glucoside, peonidin 3-*O*-glucoside (HPLC-grade) were acquired from Sigma-Aldrich (Germany). Tyrosinase from mushroom (lyophilized powder, ≥1000 unit/mg solid), 3,4-dihydroxy-l-phenylalanine (DOPA), α-glucosidase from *Saccharomyces cerevisiae* (type I, lyophilized powder, ≥10 units/mg protein), *p*-nitrophenyl-α-d-glucopyranoside, α-amylase from porcine pancreas (type I-A, 700–1400 units/mg protein), sodium phosphate buffer solution (PBS), starch solution, dinitrosalicylic acid (DNS), pancreatin lipase (111.5 units/ mg protein), *p*-nitrophenyl palmitat, arabic gum, Triton X-100 were purchased from Sigma Aldrich (Steinheim am Albuch, Germany). All chemicals and reagents used in the experiments were of analytical grade.

### Yeast strain

Lyophilised bakery yeast, *Saccharomyces cerevisiae* (Puratos, Andenne, Belgium) was reactivated, by stationary cultivation in sterile malt must, during 24 h at room temperature. A concentration of 5x10^7^ CFU/mL fermentative medium, of activate culture was used as the vegetative inoculum both in multiplication and alcoholic fermentation samples.

### Experimental design

Response Surface Methodology (RSM) was used to optimize the anthocyanins extraction conditions from PCF. A four-factor, three-level Box-Behnken design (BBD) with three replicates in the central points was employed, where time (*X_1_*), temperature (*X_2_*), liquid/solid ratio (*X_3_*) and ethanol concentration (*X_4_*) were chosen as independent variables. In all the concentrations of ethanol used in the experiment, 1 N HCL was added at a ratio of 9:1 (v/v), however, throughout the manuscript, only the term ethanol concentration was used. About 1 g of purple corn flour was weighed and mixed with a specific volume of solvent at a specific concentration at a specific liquid/solid ratio, incubated a specific temperature and time as shown in Table 1 **(**Supplementary material**)**. Further, the samples were separated by centrifugation at 6000x*g* for 15 min, whereas the obtained supernatant was collected and analyzed for total monomeric anthocyanins content (TAC). A total of 27 experiments were carried out, the experimental data for the response (**Table 2 in** Supplementary material) were fitted using a second-order polynomial model (Eq. (2)):

(2) $$Y=\beta_{0}+\sum_{i=1}^{k}\beta_{i}x_{i}+\sum_{i=1}^{k}\beta_{\mathrm{ii}}x_{i}^{2}+\sum_{j}\sum_{i=2}^{k}\beta_{\mathrm{ij}}x_{i}x_{j}$$

where Y is the response, $x_{i}$, $x_{j}$ are the independent variables (with *i* and *j* ranging between 1 to k), *β*_0_ is a constant, whereas *βi*, *βii*, and *βij* are the regression coefficients of linear, quadratic and interaction terms, *k* is the number of independent variables.

**Table 1:** Experimental data of the validation of predicted values for total anthocyanins content (TAC) at optimal extraction conditions.

**Dependent variable**	**Predicted value**	**95 % Confidence intervals**	**Experimental value**	**CV** **(%)**	**Error (%)**
TAC (mg C3G/g DW)	13.77	12.19 – 15.36	14.04 ± 0.02	1.08	1.95

### Optimization and validation of the extraction conditions

The optimal extraction conditions of TAC were estimated as reported previously by Dumitrașcu et al. (2019), using the response optimizer function available in the Minitab 18 software. Validation experiments were performed in triplicate under the optimized conditions, the experimental results being compared to the model predicted values based on coefficient of variation (CV, %) and percentage errors.

### Total anthocyanin’s concentration determination

The total anthocyanin quantification described Lao and Giusti (2015) was used. The supernatant obtained as described at section 2.4 was subjected to the operation of measuring the absorbance of the spectrophotometer (UV–vis Spectrophotometer with double beam with data analysis software, JENWAY) at a wavelength of 535 nm. The measurement was made in triplicate. The final formula for total anthocyanins has been reduced to Eq. (1):

(1) $$Total\;anthocyanins\;\left(mg/g\right)=\left(\frac{\mathrm{AxDFxV}}{98,2xX}\right)$$

where: 98.2 - unit conversions into consideration, *A* - absorbance of sample at 535 nm, *DF* - dilution factor, *V* – the anthocyanin’s extract volume obtained after extraction (mL); *X* - the weight of PCF used in extraction experiments (g) (Lao & Giusti, 2015).

### DPPH radical scavenging activity

The DPPH (1,1-diphenyl-2-picrylhydrazyl) radical scavenging activity was performed as reported previously (Slavu (Ursu) et al. 2020). Briefly, 0.1 mL of extract solution (1 mg/mL) was mixed with 2.9 mL of 0.1 mM DPPH in methanol solution and incubated for 60 min in dark. The absorbance was measured at 515 nm using a spectrophotometer (Jenway, UK). The antioxidant activity was expressed as mMol Trolox equivalents/g DW according to the equation obtained from the standard graph using Trolox as standard.

### Total flavonoid content in the extract

The total flavonoids content (TFC) were determined by using aluminum trichloride method, respectively, as reported by Meziant et al. (2021). The TFC content was expressed in mg catechin equivalents (CE)/g DW.

### Chromatographic analysis of the extract

The separation and identification of the bioactive compounds from the PCF optimized extract was carried out by an Agilent 1200 HPLC system equipped with autosampler, degasser, quaternary pump system, multi-wavelength detector (MWD) and column thermostat (Agilent Technologies, Santa Clara, CA, USA). A Synergi Max-RP-80 Å column (250 × 4.6 mm, 4 µm particle size, Phenomenex, Torrance, CA, USA) was used for polyphenolic compounds separation. For the separation of the flavonoids and polyphenolic compounds from PCF extract, the solvent A contained ultrapure water:acetonitrile:formic acid in a ratio of 87:3:10 (*v*/*v*/*v*), whereas solvent B contained ultrapure water:acetonitrile:formic acid in a ratio of 40:50:10 (*v*/*v*/*v*) (Antoniolli et al. 2015). These solvents were flushed into the system with a flow rate of 0.5 mL/min at 30℃ using an injection volume of 20 µL, using the following gradient: 0 min – 94 % A; 20 min – 80 % A; 35 min – 60 % A, 40 min – 40 % A, 45 min – 10 % A. The method runtime was 80 min, with detection at wavelength at 280 nm and 320 nm. For anthocyanin’s detection the wavelength was at 520 nm in the previously mentioned conditions, using a flow rate of 1.0 mL/min and a method runtime of 50 min. For the identification of the bioactive compounds from the PCF extract, a comparison of the retention times of the peaks with those obtained for standard solutions was used, followed by quantification using external calibration curves using the peak area. Data acquisition was made by Chemstation software, version B.04.03 [16] (Agilent Technologies, Santa Clara, CA, USA). Results were expressed in mg/100 g DW extract.

### Enzyme inhibitory activities of validated extract

The optimized extract was dissolved in 0.1 M PBS at pH 6.9 at a concentration of 5 mg/mL, and from this concentration, serial dilutions were performed. The results for enzymes inhibitory assays are expressed as a mean of three replicates and given as 50 % inhibition concentration (IC50) calculated from the linear regression of inhibitory activities (%) versus extract concentrations, using the following equation for inhibitory activity (Eq. (3)):

(3) $$Inhibitoryeffect\left(\%\right)=\frac{A_{c}-A_{s}}{A_{c}}x100$$

where: Ac = Absorbance of the control, As = Absorbance of the sample. In all the experiments, the blank sample absorbance’s (the mixture containing extract and substrate solution without the enzyme replaced by the buffer) were recorded and subtracted from the absorbance.

#### Tyrosinase inhibitory activity

The tyrosinase (from *Agaricus bisporus*, ≥1000 units/mg solid) inhibitory ability of the optimized extract was performed according to the protocol described by Meziant et colab. (2021), by mixing a volume of 0.5 mL of extract solution with 0.8 mL of tyrosinase solution (46 U/mL), 2.0 mL of PBS (0.1 M, pH 6.9) and incubated for 15 min at 37 °C. Then, 1 mL of l-DOPA (2.5 mM in 0.1 M PBS at pH 6.9) was added to initiate the reaction, followed by incubation for 10 min at 37 °C and absorbance reading at 492 nm (Jenway, UK). The tyrosinase inhibitory activity was expressed as mg/mL, using kojic acid as standard.

#### α-Amylase inhibitory activity

For the α-amylase inhibitory assay, a volume of 100 μL of samples was added over 100 μL of α-amylase enzyme solution (1 mg/mL PBS) and left at room temperature for 10 min. Further, a volume of 100 μL of 1 % starch solution was added and incubated at 37° C for 20 min. Finally, 0.2 mL of dinitro-salicylic acid was added and the samples were kept in a water bath at 98 °C for 10 min. The final mixture was diluted in 2 mL of distilled water and the absorbance was read at 540 nm. The α-amylase inhibitory ability of the PCF extract was expressed as IC50 (mg/mL), using acarbose as standard drug.

#### α-Glucosidase inhibitory activity

The method described by Meziant et al. (2021) was used to evaluate the potential inhibitory effect of the extract on α-glucosidase, with minor changes. In brief, 100 μL of the extract were mixed with 20 μL of the enzyme solution (1 mg/mL in PBS buffer 0.1 M, pH 6.9), then incubated for 10 min at 37 °C. After incubation, a volume of 40 μL of 5 mM *p*-nitrophenyl- *α*-d-glucopyranoside in 0.1 M PBS (pH 6.9) was added, followed by incubation at 37 °C for 20 min, followed by reading the absorbance at 405 nm. The α-glucosidase inhibitory ability of the PCF extract was expressed as IC50 (mg/mL), using acarbose as standard.

#### *Lipase inhibitory* activity

The method involved the hydrolysis of *p*-nitrophenyl palmitate to *p*-nitrophenol at 400 nm was used to evaluate the effect of PCF extract on lipase activity. In brief, lipase solution (1.0 mg/mL in PBS, pH 7.0) was mixed with the PCF extract at concentration ranging from 0.9 to 7.2 mg/mL) and pre-incubated on ice for 5 min. The reaction mixture contained 330 µL of PBS 0.1 M (pH 7.0) supplemented with 0.6 % (v/v) Triton X-100 and 0.15 % (w/v) arabic gum, and 20 µL of 10 mM *p*-nitrophenyl palmitate. The enzymatic reaction started by adding 50 µL of the lipase/PCF extract solution into the substrate mixture, and incubated at 37 °C for 20 min. The lipase inhibitory activity was expressed as IC50 (mg/mL), using orlistat as standard drug.

### Metabolic activity of the yeast

#### The multiplication and viability of yeast cells

In order to assess the multiplication yeast cells stage, three samples were performed, in which PCF concentrated extract were added to 100 mL of the sterile medium (liquid malt must, LMM), in different concentrations, as follows: 0.3 % (V1), 0.6 % (V2) and 1.2 % (V3), respectively. The yeast population density was analyzed by a direct method of cell counting using the Thoma chamber for 72 h (0 h, 8 h, 12 h, 24 h, 48 h, 72 h) at 25 °C, under stationary cultivation conditions. Yeast viability assay was examined by microscopy in the presence of the blue methylene indicator, based on the viable capacity of reducing the redox indicator from the blue oxidated form (blue) to the reduced form a leuco-derivative (colorless). A control test without extract addition was performed (MV).

#### Dynamics of alcoholic fermentation

The fermentation dynamics of yeast cells was determined using the same concentration of the PCF extract as for multiplication study, the samples being coded with F. The fermentation process was analyzed for 144 h (0 h, 8 h, 12 h, 24 h, 48 h, 72 h, 144 h) at 25 °C under anaerobic conditions at stationary cultivation. The inoculum concentration added for both determinations was 0.32 mL for each sample. A control test without extract was performed (MF).

### Statistical analysis

The results are expressed as mean values. Stepwise regression analysis was performed using Minitab 18 Software. The statistical significance of the polynomial equation (based on *p* value) was evaluated by using ANOVA, as well as the regression coefficients of individual linear and quadratic terms. The accuracy and validity of the model were evaluated in terms of coefficient of determination (*R^2^)*, whereas the lack of fit test and the *F*-test were considered significant at *p*-value < 0.05.

## Results and discussion

### The effect of independent variables on the recovery of anthocyanin’s from purple corn flour

In this study, a three-level, four-factor BBD was performed to evaluate the effect of various independent variables on the recovery of TAC from PCF. The experimental data were collected in **Table 2 (**Supplementary material**)**. The ANOVA parameters presented in **Table 3 (**Supplementary material**)** indicated that second order regression was significant (p < 0.05) and quadratic polynomial model fitted to the experimental results. The lack of fit test did not show a significant influence, indicating a high accuracy of the model for the prediction of TAC. The determination coefficient *R^2^* suggested that the model is able to explain 79 % of TAC variation.

From **Table 3 (**Supplementary material**)** it can be seen that at linear terms, temperature, liquid/solid ratio, and ethanol concentration presented a significant effect on TAC, whereas at quadratic terms only liquid/solid ratio displayed a significant effect. The regression model resulted after eliminating the insignificant terms was expressed as follows:

$$\mathrm{TAC}=0.45+0.14X_{2}--0.16X_{3}+0.39X_{4}--0.003X_{4}^{2}$$

where: *X_2_* is the temperature (°C), *X_3_* is the liquid/solid ratio (mL/g), and *X_4_* is ethanol concentration (%). From regression equation it can be seen a positive effect exerted by temperature and ethanol concentration on the recovery of TAC, and a negative effect exerted by liquid/solid ratio on both linear and quadratic terms.

In order to obtain high extraction yields, increased attention has to be given to parameters interval chosen for extraction. Liquid-solid ratio plays a significant effect on the yield of bioactive compounds extraction. For example, in a recent review, Cristianini & Sanchez (2020) reported that two of the most significant variables in the anthocyanins extraction from purple corn cob are solid/liquid ratio and pH. In a recent study conducted by Rafael (2017), the author used a liquid/solid ratio of 20 and reported an anthocyanins content of 7.43 mg C3G/g.

**Fig. 1:**
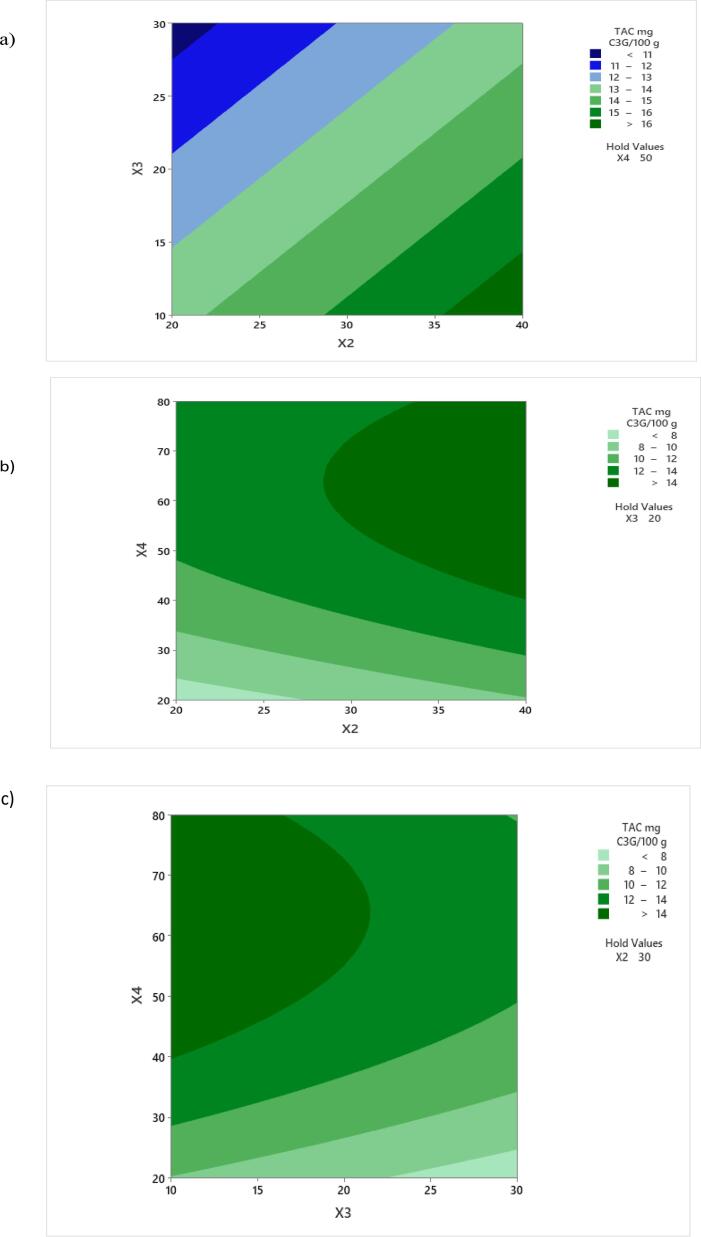
Contour plots of anthocyanin content from purple corn flour with respect to *X_2_* -temperature and *X_3_* - liquid/solid ratio (a); *X_2_* - temperature and *X_4_* - ethanol concentration (b); *X_3_* liquid/solid ratio and *X_4_* - ethanol concentration (c). (For interpretation of the references to colour in this figure legend, the reader is referred to the web version of this article.)

Besides solid–liquid ratio, temperature is another important variable that can affect the extraction procedure. The diffusion of bioactive compounds, mainly anthocyanins can be accelerated at higher temperature, however a temperature above 40 °C can result in anthocyanins degradation as revealed previously by Timberlake (2009). In order to demonstrate the relationship between the independent and dependent variables and determine their optimal levels contour plots were also depicted (Fig. 1). The plots were obtained by plotting the response of TAC using the *z*-axis against two independent variables while maintaining the other independent variable at a constant level. The effect of the solid–liquid ratio and temperature on TAC at constant ethanol concentration of 50 % is shown in Fig. 1**a**. The anthocyanin content increased with increasing temperature and decreasing the liquid/solid ratio. Blackhall et al. (2018) showed that anthocyanins content increased when using a liquid/solid ratio of up to 10 mL/g, similar results being reported by Dumitrașcu et al. (2019), a further increase resulted in the decrease of anthocyanins recovery.

In Fig. 1**b** is depicted the effect of temperature and ethanol concentration on TAC extraction at constant liquid/solid ratio of 10 mL/g. It can be seen that TAC increased with increasing of both temperature and ethanol concentration and reached a maximum when using an ethanol concentration of 80 % and an extraction temperature of 40 °C. On the other hand, when considering the effect of solid/liquid ratio and ethanol concentration on anthocyanin extraction (Fig. 1**c**), it can be seen that higher values of TAC are obtained when ethanol concentration ranged between 40 and 80 %, whereas liquid/solid ratio ranged between 10 and 20 mL/g. Ali et al. (2018) reported that using solvents with similar characteristics to the matrix favors the solubilization of compounds from the matrix.

### Process validation of the predicted value

The optimum extraction conditions proposed by the model, based on the desired function method, were performed to validate the model equation. The optimum conditions for the maximum recovery of TAC were: extraction time of 5 h, temperature of 39˚C, liquid/solid ratio of 30 mL/g and ethanol concentration of 73 %. In these conditions, the maximum response predicted for TAC was of 13.77 mg C3G/g dry weight (DW). Experimental results obtained under the optimum extraction conditions predicted for TAC confirmed the validation extraction model (Table 1). The validation is also supported by percentage errors that was lower than 10 % and by low variation coefficient (CV). Hwang et al. (2016) studied the extraction of bioactive compounds from purple maize (*Zea mays* L.), and found that the highest recovery of anthocyanins was obtained when using the following extraction conditions: a temperature of 40° C, extraction time of 8 h, a solid–liquid ratio of 33 % and a solvent volume of 1:15. These authors suggested an extraction yield for TAC of 42.28 mg C3G/100 g.

### Phytochemical profile of the validated extract

**Fig. 2:**
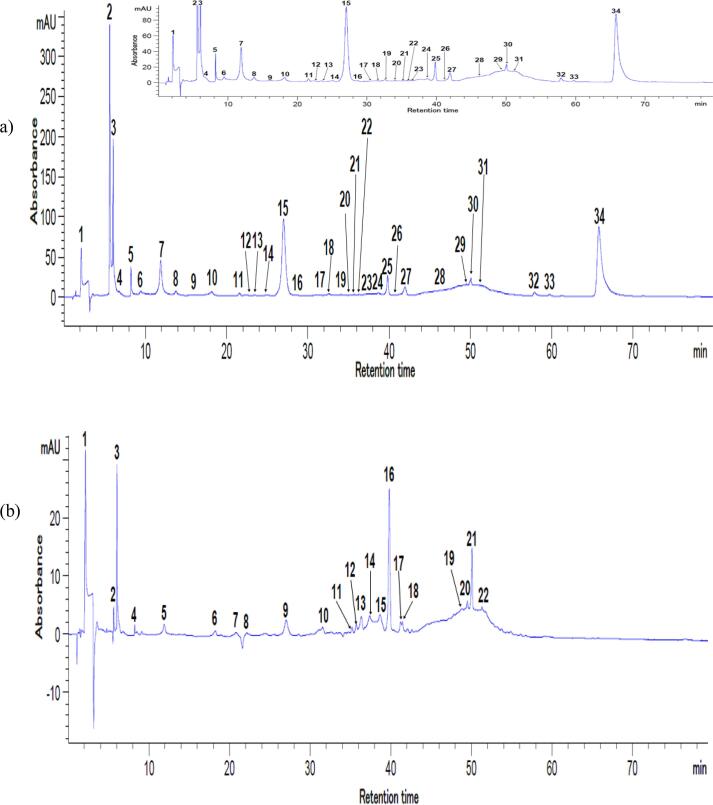
HPLC chromatograms for the flavonoids and polyphenols from purple corn flour extract at 280 nm (a) and 320 nm (b). Peaks’ identification: (a) 2 – Gallic acid, 4 – Caffeic acid, 8 – Vanillic acid, 25 – Myricetin, 28 – Quercetin 3-β-d-glucoside, 31 – Kaempferol, 1, 3, 5 – 7, 9 – 24, 26, 27, 29, 30, 32 – 34 – unidentified peaks; (b) 2 – Gallic acid, 7 – p-coumaric acid, 16 – Myricetin, 22 – Kaempferol, 1, 3 – 6, 8 – 15, 17 – 21– unidentified peaks. (For interpretation of the references to colour in this figure legend, the reader is referred to the web version of this article.)

**Fig. 3:**
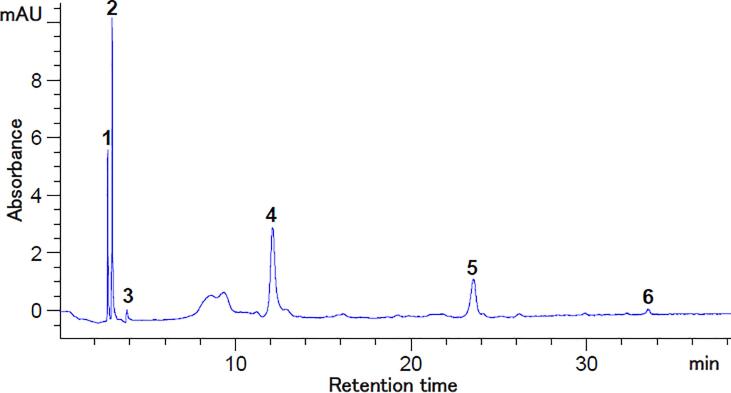
HPLC chromatogram for the anthocyanins from purple corn extract at 520 nm. Peaks’ identification: 4 – Cyanidin 3-*O*-glucoside, 6 – Malvidin chloride, 1 – 3, 5 – unidentified peaks. (For interpretation of the references to colour in this figure legend, the reader is referred to the web version of this article.)

In order to provide additional information regarding the phytochemical content of the anthocyanins enriched extract, the flavonoid contents and antioxidant activity were determined. The TFC content of the extract was 1.37 ± 0.05 mg CE/g DW, yielding an antioxidant activity of 55.61 ± 0.25 mM Trolox/g DW. Rajha et al. (2020) reported a TPC content in purple corn of 78 mg GAE/g DW and a TAC of 36 mg C3G/g DW. The chromatographic profile of the optimized extract is showed in Fig. 2. From Fig. 2 it can be observed that using different wavelength allowed to separate 34 compounds, whereas only gallic acid (349.00 ± 4.39 mg/100 g DW), *p*-coumaric acid (260.01 ± 1.90 mg/100 g DW), caffeic acid (19.73 ± 1.89 mg/100 g DW), and vanillic acid (245.46 ± 8.00 mg/100 g DW) were identified and quantified. The main flavonoids identified were myricetin (722.89 ± 2.70 mg/100 g DW), quercetin 3-β-d-glucoside (688.16 ± 5.00 mg/100 g DW) and kaempferol (512.72 ± 9.99 mg/100 g DW). Six anthocyanin’s were separated (Fig. 3**)** namely cyanidin 3-*O*-glucoside (395.66 ± 1.26 mg/100 g DW) and malvidin chloride (159.30 ± 0.04 mg/100 g DW). Hong et al. (2020) identified eighteen different anthocyanin compounds, respectively cyanidin-, peonidin-, and pelargonidin-glucosides, in pigmented sweetcorn and maize, when the extraction was carried out with 80 % (v/v) methanol acidified with 0.1 N HCl, using a liquid/solid ratio of 7.76 mL/g. These authors reported a content in cyanidin 3-*O*-glucoside ranging from 72.4 to 137.1 mg/100 FW in purple-pericarp sweetcorn, emphasizing the major impact of the extraction parameters and corn flour’s composition on specific bioactive compounds.

### The inhibitory activity of the optimized extract

The tyrosinase activity is associated to diseases such as hyperpigmentation, melanoma and Parkinson. Therefore, potential inhibitors of tyrosinase activity can represent an alternative for these diseases treatment, such as phenolic acids, flavonoids and anthocyanins (Koyu et al. 2018). In this case, the extract inhibitory capacity was compared with kojic acid, as a reference drug. The IC50 for the PCF optimized extract was 1.15 ± 0.01 mg/mL. The references value for kojic acid was 1.12 ± 0.14 mg/mL, therefore suggesting that the extract had the same effect as the reference drug.

Koyu et al. (2018) determined IC50 value for tyrosinase inhibitory activity of 166 µg/ml for an optimized extract *Morus nigra* fruits. Meziant et al. (2021) suggested that the flavonoid-rich obtained from different fig cultivar (*Ficus carica* L.) peel extracts showed tyrosinase inhibition with IC 50 values between 95.08 and 447.49 μg/mL. It has been reported that quercetin may inhibit tyrosinase activity (Fan et al. 2017), whereas cinnamic acid has been proved to possess an inhibitory impact on the expression of melanin related enzymes (Garcia-Jimenez et al., 2018). Yu et al. (2019) suggested that quercetin, isorhamnetin and gallic acid can inhibit tyrosinase activity, whereas 4-hydroxycinnamic and ferulic acid acids can cause weak tyrosinase inhibitory ability. However, given the structural particularities of tyrosinase (Yu et al. 2019), it may be possible that the inhibitory effect of PCF extract on tyrosinase to be caused by a joint effect of the polyphenolic compounds.

Different strategies are been using in inhibition of enzymes involved in carbohydrate digestion, in order to control the hyperglycemia, diabetes mellitus and lipid disorders, through α-glucosidase and α-amylase inhibition with acarbose. In our study, the potential of PCF extract to inhibit the α-glucosidase and α-amylase, and therefore to test the potential use as alternative for drug administration. The IC50 values for α-glucosidase and α-amylase were 2.30 ± 0.14 mg/mL and 0.65 ± 0.01 mg/mL, whereas the corresponding values for acarbose were 3.79 ± 0.62 mg/mL and 2.98 ± 0.13 mg/mL, respectively. The corresponding IC50 value for lipase inhibition was 0.20 ± 0.03 mg/mL, with a corresponding value for orlistat of 1.23 ± 0.09 mg/mL. It has been suggested that the flavonoid-rich extracts were more potent inhibitors of digestive enzymes than anthocyanins-rich extracts (Kim et al., 2020, Siegień et al., 2021). Therefore, it is fair to consider that the content of flavonoids in PCF extract may be responsible for the inhibitory activity, although a joint effect is also possible. Previous studied suggested that flavonoids possessed excellent inhibitory effects on α-glucosidase (Semaan et al. 2018). Quercetin derivatives and kaempferol are the most predominant flavonoids in the PCF extract, showing an excellent inhibitory effect on α-glucosidase, α-amylase and lipase, in good agreement with Hamed et al. (2021). From the obtained results, it seems that PCF extract is more effective in inhibiting the lipase, followed by α-amylase*,* tyrosinase and α-glucosidase.

### The effect of purple corn polyphenols on yeast cells multiplication dynamics

Given the variety of nutrients, purple corn can be used in different fermentation products to produce bioethanol, biosurfactants, aminoacids, antibiotics, organic acid, etc. (Zhang et al. 2021), or as raw material or adjuvants in bakeries, to develop low or free gluten products. The significant development of biotechnology tools, cornstarch is used as the main material in bioethanol production, due to its capacity to be easily converted into simple sugars by hydrolysis, followed by *Saccharomyces cerevisiae* fermentation to produce ethanol. The starch granule is found in abundance in the endosperm of the corn kernel, being developed by successive layering of amorphous and crystalline starch molecules and surrounded by matrixes and protein bodies (Ostrander, 2015). In our study, the PCF extract was tested as adjuvant in fermentation culture medium of bakery yeasts, in order to test the effect on metabolic activity of yeast, in terms of multiplication, viability of cells and dynamics of alcoholic fermentation.

**Fig. 4:**
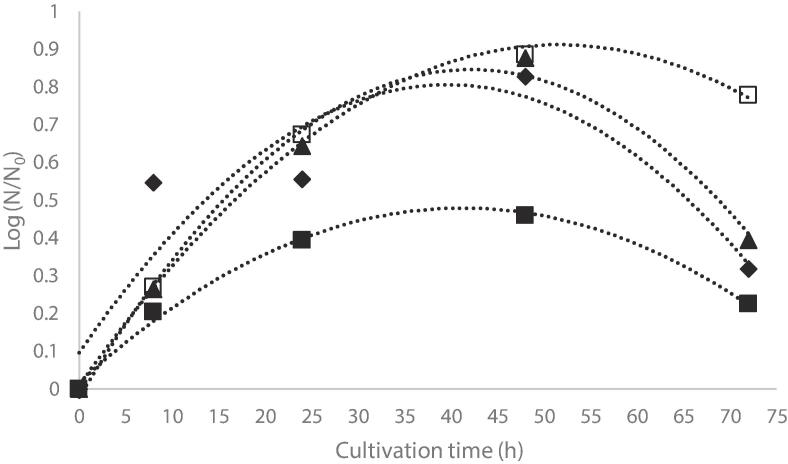
Dynamics of the yeast’s multiplication by cultivation in liquid medium supplemented with purple corn flour extract: (■) control sample – MV; (♦) sample with 0.3% extract (V1) (▲) sample with 0.6% extract (V2); (□) sample with 1.2% extract (V3).

From Fig. 4, it can be observed that the highest multiplication rate was obtained after 48 h of cultivation, being correlated with the highest amount of added PCF extract (1.2 mg/100 mL), compared to the control sample. The generation number (*n*) of yeast cells significant increases from 2.88 ± 0.76 in case of control samples to 6.72 ± 0.88 in case of V1, 7.52 ± 0.16 and 7.68 ± 0.56 in case of V2 and V3, respectively. The growth rate (*v*) significantly increases from 5.23 ± 0.97 h^−1^ in case of MV to 1.91 ± 0.11 h^−1^ to V1 and 1.44 h^−1^ and 1.40 h^−1^ in case of V2 and V3, respectively. Therefore, it can be appreciated that no significant differences were observed when considering added PCF extracts with concentrations ranging from 0.6 to 1.2 %. Significant effect of the added extract was observed when considering the generation time, the time interval for yeast cells to double its population being of 1.81 ± 0.23 h for MV sample, and significantly decreases to 0.28 ± 0.01 h for V1, 0.19 ± 0.01 h for V2 and 0.18 ± 0.01 h for V3. From Fig. 4, it can be observed that cells autolysis started after 48 of cultivation, reaching a maximum after 72 h. The results showed that in the absence of PCF extract, the autolysis was faster, the percentage of autolysed cells with respect to the total number of cells in the culture is visibly reduced after 72 h of cultivation in the control samples (8.40x10^7^ CFU/mL), when compared with V1 (10.40x10^7^ CFU/mL), V2 (12.40x10^7^ CFU/mL), whereas the highest number of cells were found in V3 (30.00x10^7^ CFU/mL). The results support the hypothesis of the protection effect of the yeasts cell with anthocyanin extract, in a dose-dependent manner.

Our results are in good agreement with Râpeanu et al. (2008), who used polyphenolic extracts from red grapes to study the influence on the kinetic multiplication of yeast as well as on the alcoholic fermentation capacity. These authors suggested that the presence of polyphenols in fermentative medium positively influences the metabolic activity and stability of yeast cells, depending on the extract type (tartaric acid was more effective than citric acid).

### The effect of purple corn polyphenols on the dynamics of alcoholic fermentation

**Fig. 5:**
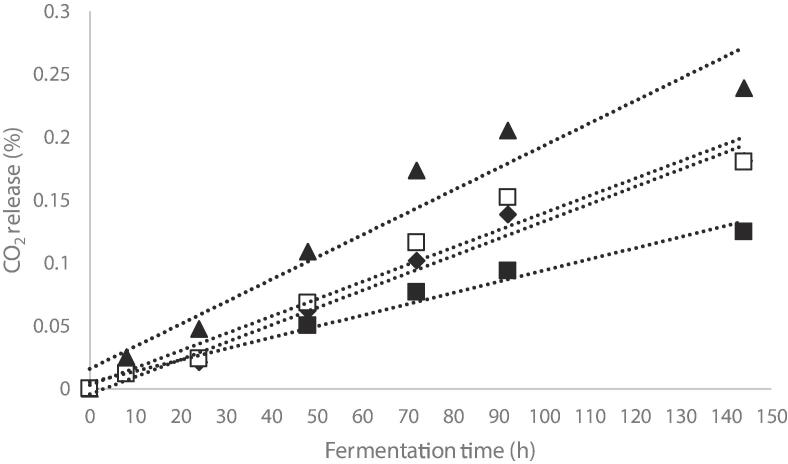
The rate of CO_2_ release in the alcoholic fermentations of liquid medium supplemented with purple corn flour extract: (■) control sample – MV; (♦) sample with 0.3% extract (V1) (▲) sample with 0.6% extract (V2); (□) sample with 1.2% extract (V3).

The fermentation dynamics was evaluated according to the CO_2_ released during 6 days. The higher total loss of CO_2_ was observed in V2 when compared with control sample VM (Fig. 5). Li et al. (2020) showed that grape-derived proanthocyanidins could act as a protector against various environmental demands for *Saccharomyces cerevisiae* during wine fermentation, resulting an increased physiological activity, fermentation efficiency and an improved wine quality.

## Conclusion

The results obtained in our study highlighted the need for phytochemical extraction optimization, in order to increase the yield of compounds in the extract. Different parameters in terms of temperature, liquid/solid ratio and ethanol concentration were tested and the experimental data were fitted using a quadratic polynomial model. In linear terms, the extraction of anthocyanins from purple corn flour depended only on temperature, liquid/solid ratio, and ethanol concentration, whereas at quadratic terms only liquid/solid ratio displayed a significant effect. The results showed o positive correlation between anthocyanin’s recovery and temperature and ethanol concentration, whereas a negative effect was exerted by liquid/solid ratio on both linear and quadratic terms. Optimal conditions for the maximum extraction yield of anthocyanin’s were established, whereas the chromatographic analysis on optimized extract revealed the highest concentration in myricetin, followed by quercetin 3-β-d-glucoside, kaempferol, cyanidin 3-*O*-glucoside and gallic acid. The extract was effective for inhibiting selected metabolic enzyme activity, such as tyrosinase, α-amylase, α-glucosidase and lipase, at lower levels than the recommended drugs. The inhibitory effect was correlated especially with the flavonoids content of the extract; however, a joint cumulative effect of the compounds on metabolic syndrome associated enzymes was suggested. Therefore, the extract showed inhibitory effect, suggesting potential antidiabetic, hypocholesterolemic and preventive effects against Parkinson’s disease and melanoma. The optimized extract was tested for the metabolic stimulation effect of *Saccharomyces cerevisiae*, the highest multiplication rate being obtained at an added concentration of 1.2 mg/100 mL. The obtained results confirm the potential use of the purple corn flour for the development of functional foods and nutraceuticals.

## CRediT authorship contribution statement

**Mioara Gabriela Slavu Ursu:** Formal analysis, Methodology, Investigation, Writing – original draft. **Ștefania Adelina Milea:** Formal analysis, Methodology, Investigation, Software, Writing – original draft. **Bogdan Păcularu-Burada:** Formal analysis, Methodology, Investigation, Software, Writing – original draft. **Loredana Dumitrașcu:** Methodology, Software, Visualization, Investigation, Validation, Writing – original draft. **Gabriela Râpeanu:** Methodology, Visualization, Investigation, Validation, Resources, Writing – original draft. **Silvius Stanciu:** Methodology, Visualization, Investigation, Validation, Resources, Writing – original draft, Funding acquisition. **Nicoleta Stănciuc:** Conceptualization, Methodology, Validation, Writing – review & editing, Supervision, Project administration.

## Declaration of Competing Interest

The authors declare that they have no known competing financial interests or personal relationships that could have appeared to influence the work reported in this paper.

## Data Availability

Data will be made available on request.
