# Supplementary material for: Optimizing of the extraction conditions for anthocyanin’s from purple corn flour (Zea mays L): Evidences on selected properties of optimized extract
Source: Food Chem X. 2022 Nov 23;17:100521. doi: 10.1016/j.fochx.2022.100521 (PMC9718927; doi:10.1016/j.fochx.2022.100521)
Supplement: Supplementary Data 1 [file mmc1.docx]

**Table 1.** Coded level of independent variables considered in the Box-Behnken design

| **Factor**  **level** | **Independent variable** | | | |
| --- | --- | --- | --- | --- |
|  | **Time (h)**  **X_1_** | **Temperature**  **(°C)**  **X_2_** | **Liquid/solid ratio (mL/g)**  **X_3_** | **Ethanol concentration**  **(%)**  **X_4_** |
| -1 | 1 | 20 | 10 | 20 |
| 0 | 3 | 30 | 20 | 50 |
| +1 | 5 | 40 | 30 | 80 |

**Table 2.** Box–Behnken design (*BBD*) design matrix with the independent variables and experimental data*^a^* for the response of total anthocyanins content (TAC) from purple corn flour

| **Run**  **Order** | ***X_1_*** | ***X_2_*** | ***X_3_*** | ***X_4_*** | **TAC**  **(mg/100 g)** |
| --- | --- | --- | --- | --- | --- |
| 1 | 3 | 30 | 10 | 50 | 14.14 |
| 2 | 1 | 30 | 20 | 20 | 6.25 |
| 3 | 1 | 40 | 20 | 50 | 15.1 |
| 4 | 5 | 30 | 20 | 20 | 7.77 |
| 5 | 3 | 20 | 20 | 80 | 11.74 |
| 6 | 1 | 30 | 30 | 50 | 11.75 |
| 7 | 3 | 40 | 20 | 80 | 11.81 |
| 8 | 5 | 40 | 20 | 50 | 15.87 |
| 9 | 1 | 30 | 10 | 50 | 15.61 |
| 10 | 5 | 30 | 20 | 80 | 13.50 |
| 11 | 3 | 40 | 10 | 50 | 19.02 |
| 12 | 3 | 30 | 20 | 50 | 13.60 |
| 13 | 3 | 30 | 10 | 20 | 8.95 |
| 14 | 3 | 30 | 20 | 50 | 13.46 |
| 15 | 3 | 20 | 20 | 20 | 7.52 |
| 16 | 3 | 30 | 30 | 20 | 8.29 |
| 17 | 5 | 30 | 30 | 50 | 13.18 |
| 18 | 3 | 40 | 20 | 20 | 11.32 |
| 19 | 3 | 20 | 30 | 50 | 9.54 |
| 20 | 3 | 30 | 30 | 80 | 13.60 |
| 21 | 3 | 30 | 10 | 80 | 14.40 |
| 22 | 3 | 40 | 30 | 50 | 10.84 |
| 23 | 5 | 20 | 20 | 50 | 12.48 |
| 24 | 3 | 30 | 20 | 50 | 15.09 |
| 25 | 3 | 20 | 10 | 50 | 13.72 |
| 26 | 1 | 30 | 20 | 80 | 15.60 |
| 27 | 1 | 20 | 20 | 50 | 11.23 |

*^a^ values are expressed as a mean value of three determination*

**Table 3.** Analysis of variance and coefficients estimate of the second order polynomial model for total anthocyanins content (TAC) from purple corn flour

| **Source** | **DF** | **Adj SS** | **Adj MS** | **F-value** | ***p*-value** |
| --- | --- | --- | --- | --- | --- |
| Model | 4 | 183.20 | 45.80 | 20.75 | 0.000 |
| Linear | 3 | 132.91 | 44.30 | 20.07 | 0.000 |
| X_2_ | 1 | 26.19 | 26.19 | 11.87 | 0.002 |
| X_3_ | 1 | 28.97 | 28.97 | 13.12 | 0.002 |
| X_4_ | 1 | 77.74 | 77.74 | 35.22 | 0.000 |
| Square | 1 | 50.29 | 50.29 | 22.78 | 0.000 |
| X_4_^2^ | 1 | 50.29 | 50.29 | 22.78 | 0.000 |
| Error | 22 | 48.56 | 2.20 |  |  |
| Lack-of-Fit | 20 | 46.93 | 2.34 | 2.87 | 0.290 |
| Pure Error | 2 | 1.63 | 0.81 |  |  |
| Total | 26 | 231.77 |  |  |  |
| R^2^ |  |  | 0.79 |  |  |
| Adj R^2^ |  |  | 0.75 |  |  |
